# Physical Exercise in the Context of Air Pollution: An Emerging Research Topic

**DOI:** 10.3389/fphys.2022.784705

**Published:** 2022-02-28

**Authors:** Yanwei You, Dizhi Wang, Jianxiu Liu, Yuquan Chen, Xindong Ma, Wenkai Li

**Affiliations:** ^1^Division of Sports Science and Physical Education, Tsinghua University, Beijing, China; ^2^Institute of Medical Information/Library, Chinese Academy of Medical Sciences and Peking Union Medical College, Beijing, China; ^3^China Table Tennis College, Shanghai University of Sport, Shanghai, China

**Keywords:** knowledge map, visualized analysis, air pollution, physical exercise, physiological mechanisms

## Abstract

Physical exercise (PE) brings physiological benefits to human health; paradoxically, exposure to air pollution (AP) is harmful. Hence, the combined effects of AP and PE are interesting issues worth exploring. The objective of this study is to review literature involved in AP-PE fields to perform a knowledge-map analysis and explore the collaborations, current hotspots, physiological applications, and future perspectives. Herein, cluster, co-citation, and co-occurrence analysis were applied using CiteSpace and VOSviewer software. The results demonstrated that AP-PE domains have been springing up and in rapid growth since the 21st century. Subsequently, active countries and institutions were identified, and the productive institutions were mainly located in USA, China, UK, Spain, and Canada. Developed countries seemed to be the major promoters. Additionally, subject analysis found that environmental science, public health, and sports medicine were the core subjects, and multidimensional communications were forming. Thereafter, a holistic presentation of reference co-citation clusters was conducted to discover the research topics and trace the development focuses. Youth, elite athletes, and rural population were regarded as the noteworthy subjects. Commuter exposure and moderate aerobic exercise represented the common research context and exercise strategy, respectively. Simultaneously, the research hotspots and application fields were elaborated by keyword co-occurrence distribution. It was noted that physiological adaptations including respiratory, cardiovascular, metabolic, and mental health were the major themes; oxidative stress and inflammatory response were the mostly referred mechanisms. Finally, several challenges were proposed, which are beneficial to promote the development of the research field. Molecular mechanisms and specific pathways are still unknown and the equilibrium points and dose-effect relationships remain to be further explored. We are highly confident that this study provides a unique perspective to systematically and comprehensively review the pieces of AP-PE research and its related physiological mechanisms for future investigations.

**Graphical Abstract:**
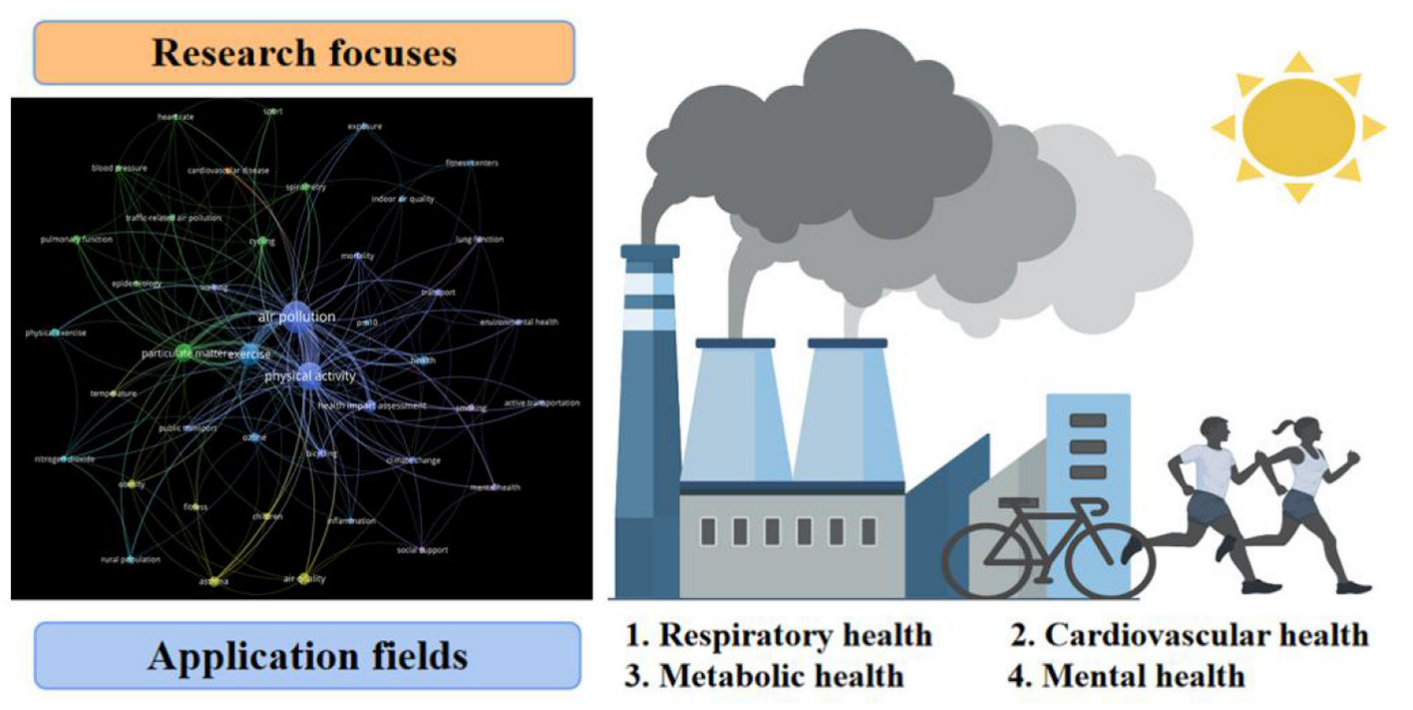
Physical exercise in air pollution: an emerging topic.

## Introduction

Air pollution (AP) occurs in sync with the progress of industrialization and urbanization. The global burden of disease assessment shows that AP has become the world's largest environmental health problem (Cohen et al., 2017) and is associated with mortality among different populations (Di et al., 2017a,b; Burnett et al., 2018). Six pollutants are designated as criteria air pollutants: particles with aerodynamic diameters under 10 and 2.5 um, ozone, sulfur dioxide, nitrogen dioxide, carbon monoxide, and lead (Suh et al., 2000). Among them, the specific particulate matter (PM) has attracted extensive attention due to its impact on several diseases, such as asthma, hypertension, diabetes, and depression (Polichetti et al., 2009; Kramer et al., 2010; Guarnieri and Balmes, 2014; Kim et al., 2016). Indeed, there is also evidence that AP has caused 4.2 million premature deaths worldwide and will become the seventh most lethal factor in the world in the next two decades (Foreman et al., 2018). As an emerging strategy to mitigate the detriment of AP to the body, exercise and physical activities play irreplaceable roles in promoting health, preventing chronic diseases, reducing disease complications, and delaying disease progressions (Haskell et al., 2007; Colberg et al., 2010; O'Donovan et al., 2010; Booth et al., 2012; Lavie et al., 2019). In this condition, a rarely known but increasingly crucial issue is the relationship between AP and its impact on exercise and physical activities. Given that physical inactivity and sedentary lifestyle itself can induce multiple chronic diseases, while outdoor exercise is regarded as a unique solution for sustaining physiological balance, improving a metabolic cycle, and enhancing immunity. These facts, in this sense, lead to a dilemma whether the positive effects of exercise could be suppressed by the ambient AP.

It is of great significance to examine the risk-benefit relationship between health benefits of physical exercise (PE) and the potential risks of the increased air pollution. First, for people living in the long-term polluted regions, they have no choice but to commute during the hustling traffic or maintain a certain intensity of physical activities no matter how serious the pollution is, so exploring the effects of exercise on health in the context of air pollution is meaningful to provide exercise suggestions (e.g., mode, duration, and intensity) and health guidelines for this targeted group. Second, with the springing up of global outdoor sports represented by major marathons, people participating in outdoor activities are also at higher risk of exposure to pollutants. Studying the safety threshold of exercise during AP exposure can not only guide city governments to organize various events, such as the Olympic Games, world, or nationwide sporting events, etc., but also help sports enthusiasts or professional athletes to prepare for these games. Finally, in view of the fact that AP itself can lead to an increase in the prevalence and mortality of chronic diseases, probing into the prevention and rehabilitation of related disease using exercise strategy during the intermittent period of AP can help to determine whether exercise can mitigate the health detriment caused by pollutant exposure.

Concerning the research on the AP, PE, and public health, a comprehensive analysis containing these three fields is lacking. In response to the increasing volume of articles on the relationship among these issues, this study aims to synthesize the accumulating knowledge in the field and provide a bird's eye view of the research using bibliometric and visualized approaches. Comparing to traditional content analysis of the literature, this strategy can provide an objective and holistic overview to discover the spatial and temporal distributions of study status, identify the major and highly-cited scholars, reveal emerging thematic frontiers, and ultimately, contribute to moving the research field forward. However, to the best of our knowledge, the systematic analysis based on the review of PE in the context of AP has not been made yet. To fill these research gaps, this study employs bibliometric mapping and the visualized method to address the following key issues: (i) to identify countries, institutions, categories, and journals that have played important roles in global AP-PE research; (ii) track current concerns and physiological fields by reviewing the nowadays' status and new efforts; and (iii) to summarize the frontiers that need to be more concerned in the AP-PE context.

Collectively, this paper's innovation lies in the following points. It is the first attempt at a knowledge-map review of AP-PE, which provides an innovative perspective to comprehensively examine the basic features of the literature and identify the hotspots. Simultaneously, building on the selection of the authoritative database, this manuscript systematically elucidates the latest international cooperation status, evolution trends, and the multi-level research on the AP-PE domain. Eventually, several priority directions of the AP-PE field are highlighted to advance the knowledge progress in this area.

In summary, this study is structured as follows: Section Methodology introduces the methodology. Subsequently, based on the knowledge map and bibliometric strategies, the characteristics of documents are presented in section Results and Discussion. In light of the reference co-citation analysis and keywords co-occurrence analysis, section Research Hotspots and Application Fields Analysis discusses the application fields and reviews the new efforts and research implications. Section Conclusion and Future Perspective concludes the study and suggests the future work.

## Methodology

### Data Acquisition and Search Strategy

Four indexes, the Science Citation Index Expanded (SCI-Expanded); the Social Sciences Citation Index (SSCI); the Arts & Humanities Citation Index (A&HCI); and the Emerging Sources Citation Index (ESCI), were selected from the Core Collection of Web of Science as the data source. Several reasons justified the selection of the WoS database in this research. As noted, the WoSCC remains as the standard database for knowledge-map-based analysis (Meho and Yang, 2007), which has been applied in a number of bibliometric studies (Mao et al., 2018; Martinho, 2019; Yan et al., 2020; You et al., 2021a,b). In addition, the WoSCC is a multidisciplinary database and includes literature on environmental science and public health emerging from distinctive research areas and disciplines published in more than 20,000 journals. Applying specialized databases such as PubMed may result in biases into the search strategy favoring medical domains. Still, it is important to note that, although other interdisciplinary databases like Scopus can provide similar coverage, it has complete citation information only from 1996 (Li et al., 2010), so the results from Scopus are still imperfect. Additionally, in terms of classification, WoS is also one step higher than Scopus in representing metadata lists. Actually, search results in WoS are not only classified by author, year, subject, and document types like Scopus but also by institutions and countries (Chadegani et al., 2013), which are more convenient for scholars to analyze the distribution of organizations. Thus, in view of the above factors, we chose the four indexes of WoSCC as the database for further analysis.

**Figure 1:**
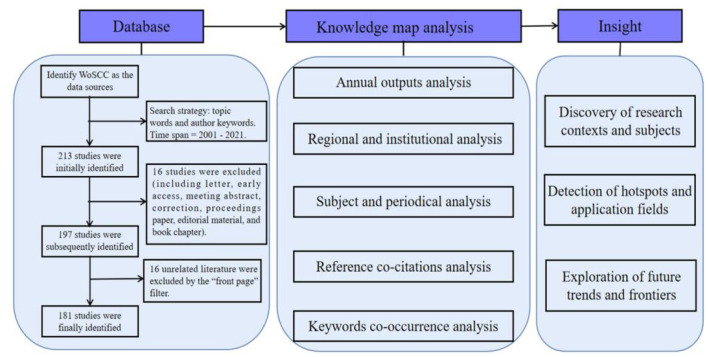
The summary of the flowchart and study design.

The following methods were conducted for search publications: [TS = (“exercise” OR “fitness” OR “sport” OR “physical exercise” OR “physical activity” OR “exercise training” OR “physical fitness” OR “aerobic exercise” OR “non-aerobic exercise” OR “interval exercise” OR “resistance exercise” OR “strength exercise” OR “breathing exercise” OR “muscle stretching exercise”] AND TS = ([“air pollution” OR “polluted air” OR “pollution air” OR “air quality” OR “indoor air pollution” OR “outdoor air pollution” OR “air pollution health” OR “air pollution cardiovascular” OR “air pollution lung” OR “air pollution asthma” OR “air pollution respiratory” OR “household air pollution” OR “ambient air pollution” OR “particulate matter” OR “ozone”)] AND [AK = (“exercise” OR “fitness” OR “sport” OR “physical exercise” OR “physical activity” OR “exercise training” OR “physical fitness” OR “aerobic exercise” OR “non-aerobic exercise” OR “interval exercise” OR “resistance exercise” OR “strength exercise” OR “breathing exercise” OR “muscle stretching exercise”] AND AK = [“air pollution” OR “polluted air” OR “pollution air” OR “air quality” OR “indoor air pollution” OR “outdoor air pollution” OR “air pollution health” OR “air pollution cardiovascular” OR “air pollution lung” OR “air pollution asthma” OR “air pollution respiratory” OR “household air pollution” OR “ambient air pollution” OR “particulate matter” OR “ozone”)], time span = 2001–2021. To avoid bias, all hits were retrieved as “full-record and cited-references” files from WoSCC on August 12, 2021 for further analysis. Referring to the previous pieces of research (Yan et al., 2020; You et al., 2021a,c), we only selected “articles or reviews” for analysis, and the language was limited to “English”; other document types and non-English articles were excluded. The reason for setting the time range in the new century was that few publications related to AP-PE were produced before year 2001. Basic information for each document was gathered into text documents, such as countries, institutions, journal sources, authors, and references. A total of 213 papers were initially found; then, the document type and language were filtered, resulting in a final number of 181 publications. The research framework is summarized in Figure 1.

### Research Methods and Analysis Tools

The research methods used in this paper were the knowledge map and bibliometrics. The knowledge-map-based methods can be used to perform visualized analysis on a certain research field, and its execution mostly involves the applications of data mining, scientific measurement, information analysis, and drawing. By mapping literature distribution and overall efforts, the knowledge map has been used widely to get more refined information related to the research itself (Rodriguez and Moreiro, 1996; Chen et al., 2005). Bibliometric overview is another widely recognized approach to evaluate topics from a library and an information science perspective (Oelrich et al., 2007; Bornmann and Leydesdorff, 2014), which can help discover knowledge flows and patterns in the structure of a field, demonstrate its scientific roots, reveal emerging thematic areas (Skute et al., 2017), and ultimately present an in-depth analysis of the knowledge domain. Combining these two strategies can provide audience with visual graphics to clearly understand the study status, frontiers, and hotspots, thereby suggesting perspectives to guide future directions.

Two java-based visualization tools, CiteSpace and VOSviewer, were used to characterize and reveal the results from bibliometric analysis. CiteSpace was developed by Professor Chen Chaomei of Drexel University, and it is mainly used to build the knowledge map (Chaomei, 2006); the software has been iterated to version 5.8.R1. In this study, the CiteSpace software was applied to visualize the network of countries and institutions, collaboration network among authors and cited authors, relationship of categories and journals (a dual-map overlay) involved in this field, and the timeline view map of references with co-citations. Noticeably, a co-citation is defined as two publications share a citation from the same third study (Small, 1973; Merig et al., 2019). VOSviewer (version 1.6.16) was applied to generate the co-occurrence of related keywords in special issues.

Additionally, a knowledge map typically features a set of points and lines to elucidate collaborations among publications (Chaomei, 2006). Different nodes are used to represent indexes like a country or region, author, institution, journal, reference, or keyword. The size of the nodes speaks on behalf of the betweenness centrality of publications, and nodes with larger size indicate higher occurrence or citation frequency (Chen et al., 2014). The betweenness centrality index in this study can be regarded as a bridge extending from earlier to more recent viewpoints, which further reflects the extent to which paths in the network go through a certain node. The values of centrality are usually standardized to the unit interval [0, 1]. Simultaneously, lines represent connections among the nodes, with stronger connections indicated by wider lines. The knowledge map represents the keywords and references with citation bursts. Occurrence bursts describe the strength of a certain theme frequency, whereas citation bursts are used to express the frequency of the reference (Chaomei, 2006). The burst of nodes was calculated with a given network through Kleinberg's algorithm (Jon, 2003). Through burst index and this kind of maps, scholars can better understand emerging trends and grasp the research hotspots.

## Results and Discussion

### Annual Outputs Analysis

**Figure 2:**
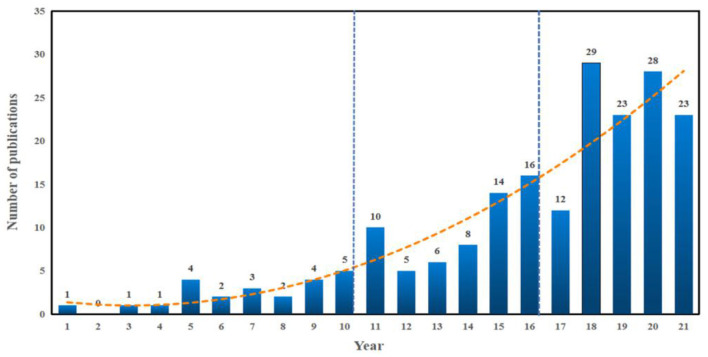
The output of publications and growth trends of pieces of air pollution-physical exercise (AP-PE) research.

A total of 181 candidate publications were finally retrieved from database WoSCC. Based on the statistical analysis, the details of annual publications since the new century are presented in Figure 2. The dynamic change in the number of publications can be used as an indicator to reveal the potential research trends. The outputs tendency can be briefly divided into the following three stages: 2001–2010, 2010–2016, 2016-present. Although the hazards of air pollutants to health have been widely reported, no further study participated in exploring the exercise activities in polluted air. In 2001, Carlisle and Sharp (2001) paid attention to exercise in the outdoor ambient air pollution, which aroused more interest in this field. While the annual outputs never exceeded five before 2010, which indicated that there are still few scholars paying attention to PE under the background of AP and the research on this domain remained stagnant in this stage. In 2010, with the enhancement of people's active health awareness, Johan de Hartog et al. (2010) proposed the question whether the health benefits of cycling outweighed the risks exposed in pollutants, and this called for more follow-up pieces of research about this issue. Subsequently, Tainio et al. (2016) in 2016 found that benefits of physical activity and active travel outweighed the harm caused by air pollution in most cases, which further strengthened people's confidence in maintaining physical activity even in a polluted environment. The number of publications on this field increased from 5 in 2010 to 16 in 2016 and has kept an overall growth trend up to now. We next drew the above results into a fitting curve and used the curve fitting method to obtain the change trend formula of the number of international AP-PE literature, as shown in formula y = −4E-06 x^3^ + 0.0843 x^2^ −0.5183 x + 1.7978. Among them, y represents the number of papers, and x represents the year. It can be estimated that the publication amount related to this topic will continue to increase in the following years.

### Regional and Institutional Analysis

**Figure 3:**
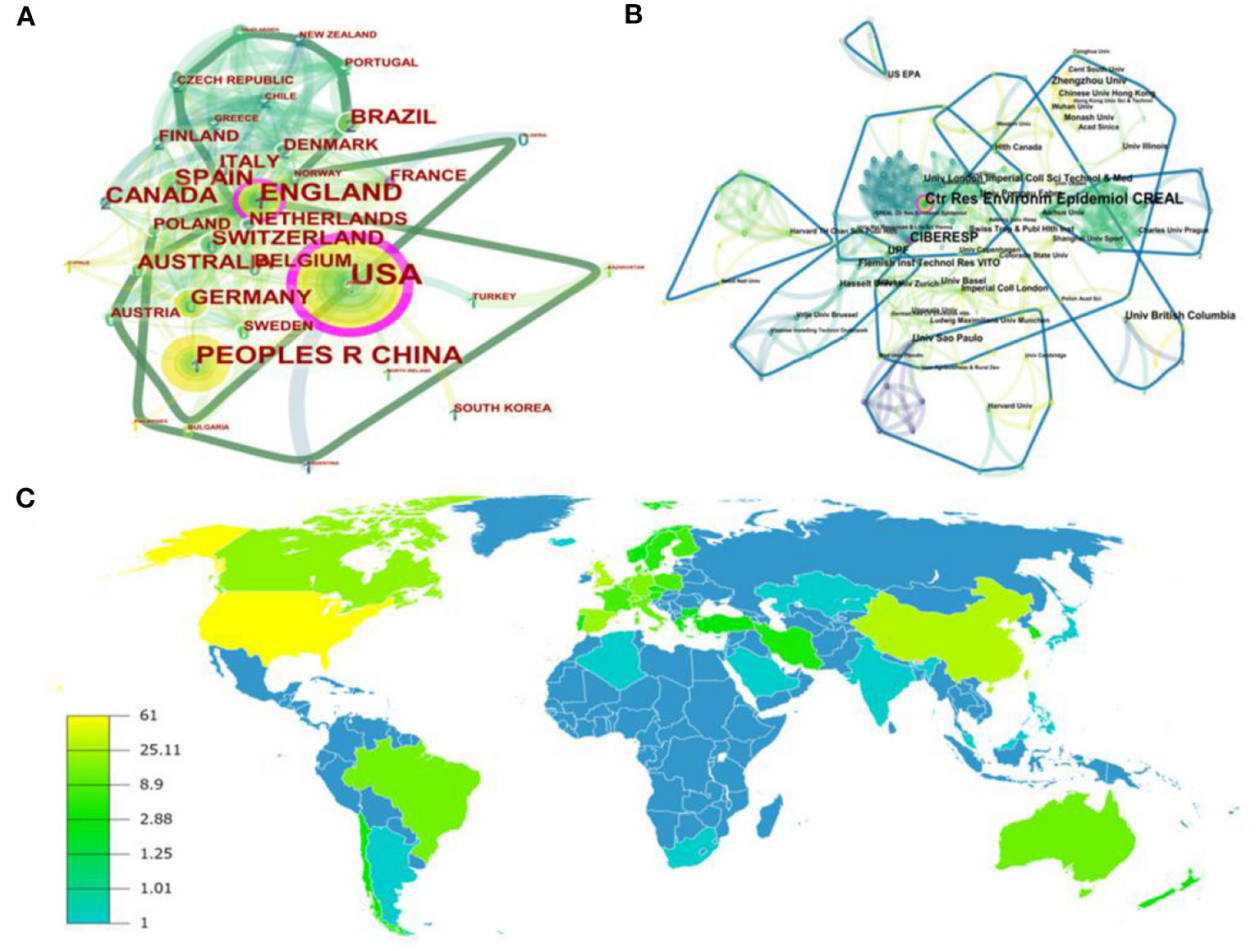
The map of countries **(A)**, institutions **(B)**, and world distribution **(C)** of publications in the AP-PE field.

Scholars from more than 40 regions and 280 institutions contributed to publications on the domain of AP-PE research. The details of the top 10 countries and institutions are shown in Table 1. The cooperation network among different countries is illustrated in Figure 3A, which describes the research status of AP-PE in multiple areas. Each point in the figure represented a country, and the size of the point reflected the number of documents in that country. The international academic collaborations were generated by CiteSpace software with 41 nodes and 228 links, which means that the total documents collected were published in 41 countries or regions, and 228 connections were built among these countries. We can observe that the mainstream cooperation relationship was divided into three groups, and the United States, the United Kingdom, and China occupied the dominant position, which conducted the majority of pieces of AP-PE research. The United States took the lead and had the highest amount of literature, 61, also the highest centrality (0.35). China and the United Kingdom were the second productive countries with 30 and 28 publications, respectively, followed by Spain (22 publications) and Canada (20 publications). The top five countries' contributions were all above 20 publications, which implied that they contributed chiefly in research achievements.

**Table 1:** Ranking of top 10 countries and institutions of pieces of air pollution-physical exercise (AP-PE) research.

**Rank**	**Country**	**Publications**	**Centrality**	**Rank**	**Institution**	**Publications**	**Centrality**
1	United States	61	0.35	1	Ctr Res Environm Epidemiol CREAL	19	0.18
2	China	30	0.09	2	Centro de Investigacion Biomedica en Red	14	0.13
3	United Kingdom	28	0.20	3	UPF	8	0.03
4	Spain	22	0.05	4	Univ British Columbia	8	0.03
5	Canada	20	0.01	5	Zhengzhou Univ	6	0.01
6	Germany	16	0.07	6	Flemish Inst Technol Res VITO	6	0.01
7	Switzerland	16	0.06	7	Univ London Imperial Coll Sci Technol & Med	6	0.01
8	Brazil	14	0.01	8	Univ São Paulo	6	0.06
9	Australia	13	0.03	9	Univ Basel	5	0.01
10	Italy	12	0.04	10	US EPA	5	0.02

When it comes to institutional participation, Figure 3B reflects the collaborations among institutions with 281 nodes and 613 links. According to the definition of links and nodes, these organizations had close ties with each other and have a considerable academic impact. Ctr Res Environm Epidemiol CREAL engaged in the most studies with 19 documents, followed by Centro de Investigacion Biomedica en Red, UPF, Univ British Columbia, with 14, 8, 8 literature, respectively. In terms of centrality, these top 10 institutions were also in a leading position. In addition, we can detect that over half of the institutions were universities, which indicated that colleges and universities played important parts in the innovation of this field. However, from the perspective of clustering, some non-university institutions seemed to have weak connections with others, while universities tended to maintain a large number of cooperative relationships.

Furthermore, the distribution of countries and regions is presented in Figure 3C. From this figure, we can find that countries and institutions in North America, western Europe, Australia, and East Asia led the way in the AP-PE domain. Consistently, most of the top 10 institutions referred in Table 1 belonged to Western nations, which also helped explain that Western nations occupied the first echelon position in the AP-PE field. It seemed that developed regions might input richer resources into environmental and health-related topics and pay more attention to physical activities in air pollution. However, as the largest developing country, China also conducted significant contributions in this research field. China faces severe challenges in controlling air pollution and preventing its potential hazards. In view of the vast territory of China, it is difficult to change the fact that some areas have suffered from industrial pollution for a long time in the short term. Hence, it is more meaningful to study the relationship between air pollution, physical activities, and their impacts to body health in these regions. In brief, western countries and institutions seemed to be the major promoters; participants from different regions all made contributions to the AP-PE field; and different organizations should strengthen cooperation to win higher achievements.

### Subject and Periodical Analysis

**Figure 4:**
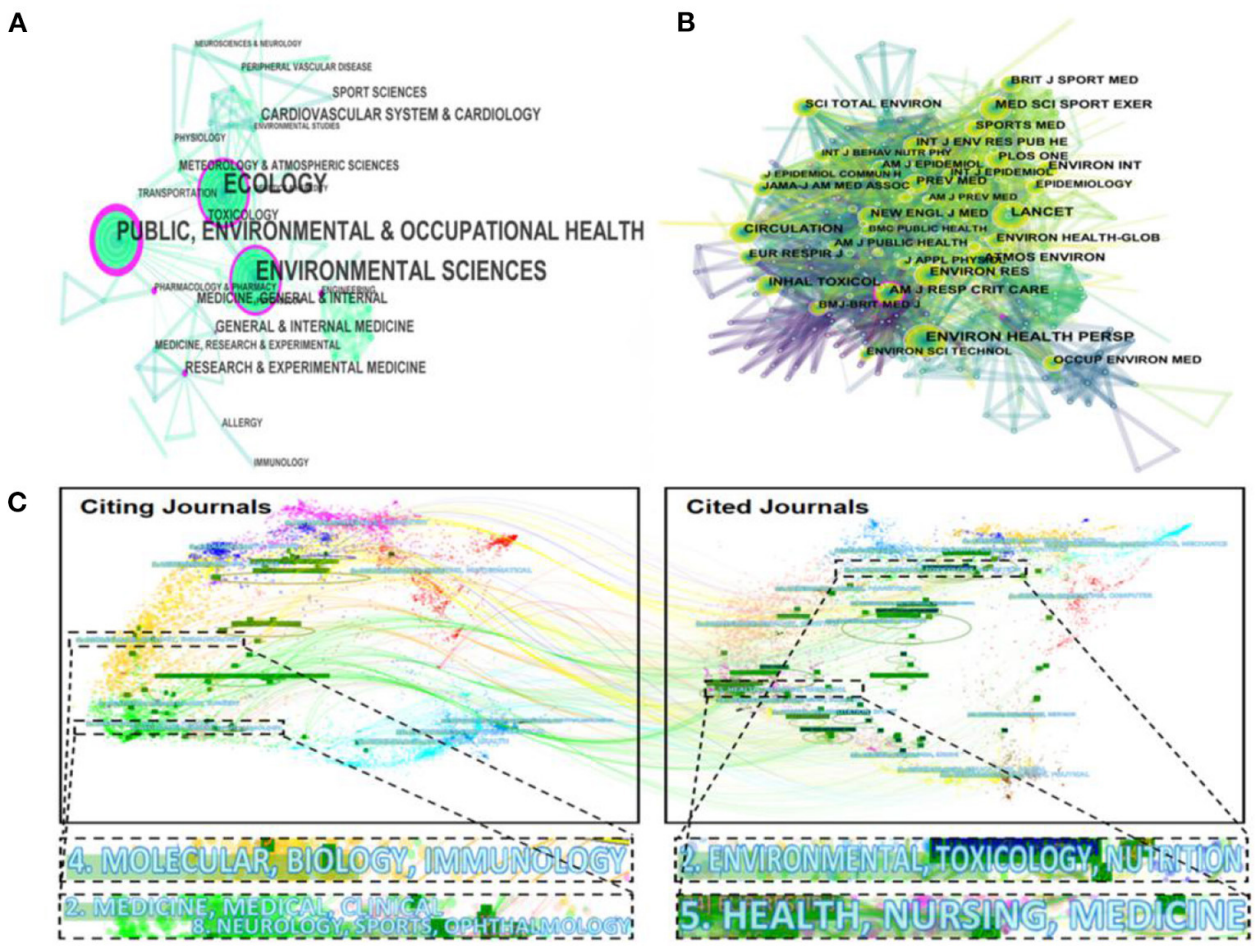
The map of disciplines **(A)**, cited-journals **(B)**, and a dual-map overlay **(C)** of publications in the AP-PE field.

Subjects involved in publishing pieces of AP-PE research are displayed in Figure 4A. Referring to the description of centrality, the discipline “Ecology,” “Public, Environmental, and Occupational Health,” and “Environmental Sciences” are surrounded by the purple rings, which indicated that they played important roles in AP-PE studies. The merged network was consisting of 71 nodes and 145 links, which illustrated that 71 sub-disciplines were involved in pieces of AP-PE research and 145 cross-paths, and collaborative networks have been established within the disciplines. Compared to previous AP literature (Sweileh et al., 2018; Dhital and Rupakheti, 2019), the disciplinary cooperation networks in the fields of AP-PE were more complex and comprehensive. It was clear that all subjects made contributions to the scientific research. Environmental science and public health seemed to be core subjects, while sports science, medicine, and toxicology worked together to enrich the horizons of the disciplines and broaden the depth of research. Importantly, as the variety of disciplines tended to be diversified, it can be seen that the AP-PE field has developed rapidly in the past decades.

Building on the analysis of subjects, we next discussed about the cited journal in the AP-PE domain. The term citing journal is defined as the journal that publishes AP-PE papers, and the cited journal is defined as the journal where the references cited in AP-PE papers are published. Figure 4B describes a panorama of cited journals. In this figure, 471 nodes and 2,939 links were built simultaneously. Among the nearly 500 journals, there were Sci Total Environ, Environ Int, Environ Res et al. in environmental science; Med Sci Sport Exer, Brit J Sport Med, Sports Med et al. in sports science; the medical field represented by Lancet, New Engl J Med, and Circulation. Several comprehensive journals, such as Plos One, were also among the abundant research scopes. Given that cited journals provided a theoretical basis for the citing journals, these diverse trajectories illustrated that the disciplinary center of the journals shifted from a single subject to multidisciplinary clusters.

Figure 4C presents a dual-map overlay of the research themes between citing journals and cited journals in the AP-PE field. This approach uses two graphs at the same time, with the citing journals on the left and the cited journals on the right. From this figure, we can find two major citation paths. The yellow paths showcased that research published in “molecular, biology, immunology” journals preferred to quote journals mostly in the domains of “environmental, toxicology, and nutrition.” The paths colored with green demonstrated that studies published in “medicine, medical, clinical,” and “neurology, sports, and ophthalmology” journals tended to cite journals primarily in the domains of “health, nursing, and medicine.” It is to be noticed that AP-PE fields are actually a comprehensive interdisciplinary as well as an emerging realm worthy to be explored. For promoting the development of physical activities in air-polluted environments, there is an urgent need for more multidimensional communications.

### Analysis of Co-cited References

Co-cited references refer to the citations of two scientific documents by one article (Chaomei, 2006). The more occurrences of co-cited documents, the closer the relationship between the two scientific documents. As time evolves, a huge structure of citation relationships has been formed among published scientific documents. In this study, we used the CiteSpace software to concretely express the relevant citation relationship network structure in the AP-PE field through the scientific knowledge map. By analyzing the clusters composed of co-cited references, we can have access to trace the roots of the scientific development and on this basis, to explore the themes and trends of AP-PE fields.

**Figure 5:**
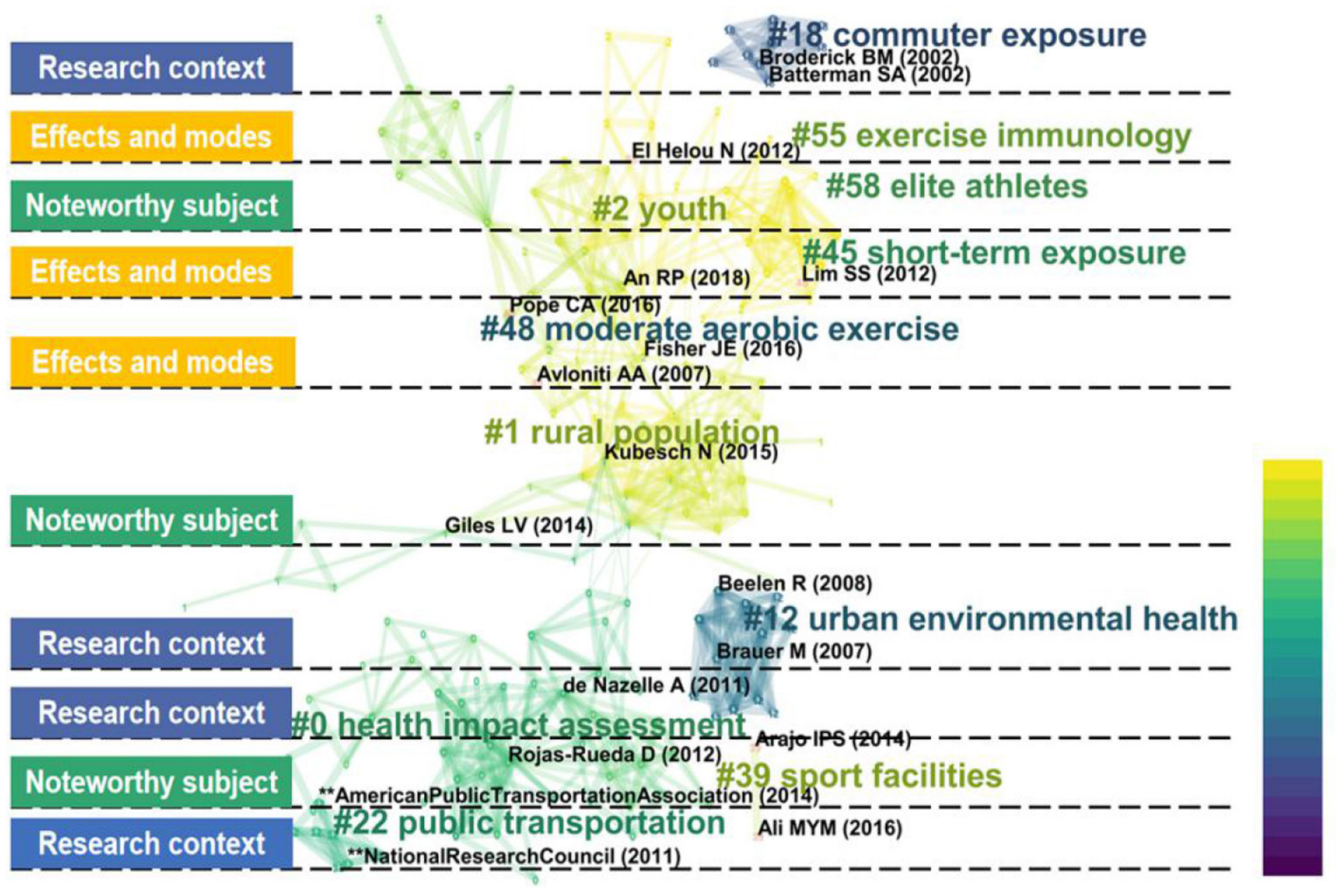
The research topics and clusters of the co-cited references in the AP-PE field.

There are three options that can be applied to create label clusters: Log-Likelihood Ratio (LLR), Latent Semantic Indexing (LSI), and Mutual Information (MI). We used the Log-Likelihood Ratio algorithm since this strategy can cover the “uniqueness and coverage” of all labels created. The research topics were divided into several clusters, which were labeled by “#,” and then their time development trajectory was described, as shown in Figure 5. The darker the clusters' color, the later the literature appeared. Here, we concluded several noteworthy topics into three classifications: “research context,” “effects and modes,” and “popular subject.” The modularity value (Q value) and weighted mean silhouette value (S value) were used to evaluate the rationality of clustering, and it is generally accepted that a Q > 0.5 cluster and S > 0.7 means that the cluster is convincing. In our clustering, the Q equaled to 0.885 and S equaled to 0.893, which further verified the rationality of the strategy.

Research context is mainly composed of four sub-clusters: commuter exposure, urban environmental health, public transportation, and health impact assessment. In recent years, “active transportation” (AT) has gradually become a popular new model, represented by walking and cycling for transportation. Motivations for the AT mode not only included traffic pollution mitigation, urban climate improvement, but also provided substantial health benefits. Unfortunately, the discussions on the benefit-risk and benefit-cost ratios of active transportation have never stopped so far. The current consensus is that AT under a lesser extent AP may be beneficial (de Nazelle et al., 2011), and, in this situation, AT can help people get rid of car dependence and increase activity levels (Lindsay et al., 2011). It is well-known that there is a dose-response relationship among pollution exposure, physical activity, and health. The greater the amount of physical activity and the lower the pollution concentration, the more likely it is to reduce the risk of morbidity and death. Therefore, there seems to be emerging interest in health impact assessment (HIA) as a strategy to evaluate health consequences. Despite the impact of potential publication bias, Mueller et al. (2015) applied the HIA method to systematically evaluate the AT mode and found that the benefits of increased PA surpassed detrimental influence of traffic incidents and air pollution exposure. In addition, Woodcock et al. (2009) estimated the relationship between AT and greenhouse gas emission reductions and suggested that mode shifts toward AT were beneficial to public health issues. Rather than traditional assessment, de Nazelle et al. (2011) first proposed a conceptual framework to further evaluate the pertinence and quantify relevant impacts. However, in view of the complexity of the active travel mode, there are still several factors, which cannot be fully quantified. Meanwhile, although modeling tools can be used to predict the relationship between vehicle emissions reduced by physical activity and AP, there are few real-world examples and lack of evidence from different regions.

In relation to research effects and modes, it can be divided into exercise immunology, short-term exposure, and moderate aerobic exercise. Immunity enhancement is one of the significant benefits that physical exercise brings to the body. A growing number of studies have proved that exercise induces considerable physiological changes in the immune system (Hoffman-Goetz and Pedersen, 1994; Nieman, 1997; Gleeson and Pyne, 2000; Pedersen and Hoffman-Goetz, 2000). Cytokines (IL-6, IL-8, IL-10, and TNF-α) (Normando et al., 2013; Bos et al., 2014; Lu et al., 2015a; Trnjar et al., 2017), inflammation-relate proteins (CRP, FeNO, CC16, CD62P, CD63, and CD40) (Rundell et al., 2007; Cutrufello et al., 2011; Normando et al., 2013; Trnjar et al., 2017), and stress hormones (including epinephrine, norepinephrine, growth hormone, b-endorphins, insulin, and cortisol) (Volek et al., 1997; Molina-Sotomayor et al., 2020) are most common indexes to estimate the variety of immune function influenced by exercise. As for the duration of the study, in view of the potential ethical approval difficulties of long-term air pollution exposure, most of the existing studies focus on the impacts of exercise and physical activities in short-term exposure to the body. Current evidence tends to show that short-term exercise on non-regular basis trainers induces a short-term inflammatory response (Kasapis and Thompson, 2005), but if with the long-term regular exercise, habits can lead to an anti-inflammatory effects, including reductions in C- reaction protein (CRP) levels (Dufaux et al., 1986). An exercise mode is another variable factor, also a quantifiable and reproducible strategy that can be modified experimentally, while the most classic and common paradigm used in current research is moderate aerobic exercise (MAE), which is also known as moderate intensity aerobic exercise (MIAE). At present, there are a number of studies that have adopted the contrast mode between high and low exercise intensities or adopted emerging exercise modes such as interval training or sprint training or the mix of both like high-intensity interval training (HIIT) (Giles et al., 2012, 2014; Marmett et al., 2021), but the overall impact of changes in the exercise mode on air pollution exposure is relatively small. Since the new exercise mode seems to be difficult for beginners or amateurs, pieces of research on the effects of different exercise patterns remain to be excavated.

When it comes to a popular subject, it is important to highlight youth, elite athletes, sports facilities, and rural population. Youth groups are in the growth period, and their respiratory system is not yet mature; hence, they are more susceptible to atmospheric particulates than adults (Gauderman et al., 2004; Chen et al., 2015; Ghozikali et al., 2018). Evidence showed that changes in respiratory tract inflammation, FeNO, etc. can be observed in school children on just 1 day of short-term exposure to air pollutants (Idavain et al., 2019). Recently, several studies have discussed the relationship between adolescent physical activities and air pollution. Results proved that outdoor activity time duration was associated with air pollution, and the longer the outdoor activity time might lead to lower lung function (Yu et al., 2017; Lovinsky-Desir et al., 2021). Except for young people, elite athletes are also the targeted groups. Different from the general population, athletes have to perform year-round training, and the climate of the training or competition sites is not selectable. It is inevitable that athletes may encounter different air pollution conditions. Early studies have been conducted on the status of particulate matter inhaled by athletes during the Athens Olympic Games and Beijing Olympic Games (Florida-James et al., 2004; Lippi et al., 2008; Fitch, 2016). Not surprisingly, several results pointed to the fact that several problematic pollutants, including oxides of nitrogen (NO_x_), PM_10_, PM_2.5_, and ozone, have a potentially deleterious impact on top-class athletes' health and athletic performance (Florida-James et al., 2004; Donnelly et al., 2016; Reche et al., 2020). Moreover, swimmers, skaters, skiers, or other groups practicing at specific sports venues are at greater risk of exposure to air pollution. Breathing airborne Teflon particles from fluorinated waxes in the confines of the indoor ski-slope may be risks to skiers (Rundell and Sue-Chu, 2013). Likewise, serious swimmers are exposed to high concentrations of trichloramines at the pool-surface level, and this kind of exposure may be continuous (Bougault and Boulet, 2012). However, it is still challenging to disentangle confounding air pollutants' effects on athletic performance under different sports circumstances. As for rural individuals, when AP is viewed in the context of low income, confounding factors can detrimentally affect their health, including physical inactivity, none medical insurance, poor nutrition, and other health risks. In relatively underdeveloped rural areas, the use of inefficient fuels and lack of sufficient ventilation led to worse air quality than other regions. Studies showed that communities with high proportions use of household solid fuel and the polluted emissions from inefficient stoves may cause ambient AP and place regions at risk for adverse health outcomes (Ward and Lange, 2010; Lim et al., 2012; Johnston et al., 2013). In a nutshell, youth groups, elite athletes, enthusiasts of special indoor sports activities, and rural population are emerging research subjects in AP-PE fields and deserve special attention.

### Analysis of Co-occurrence Keywords

**Figure 6:**
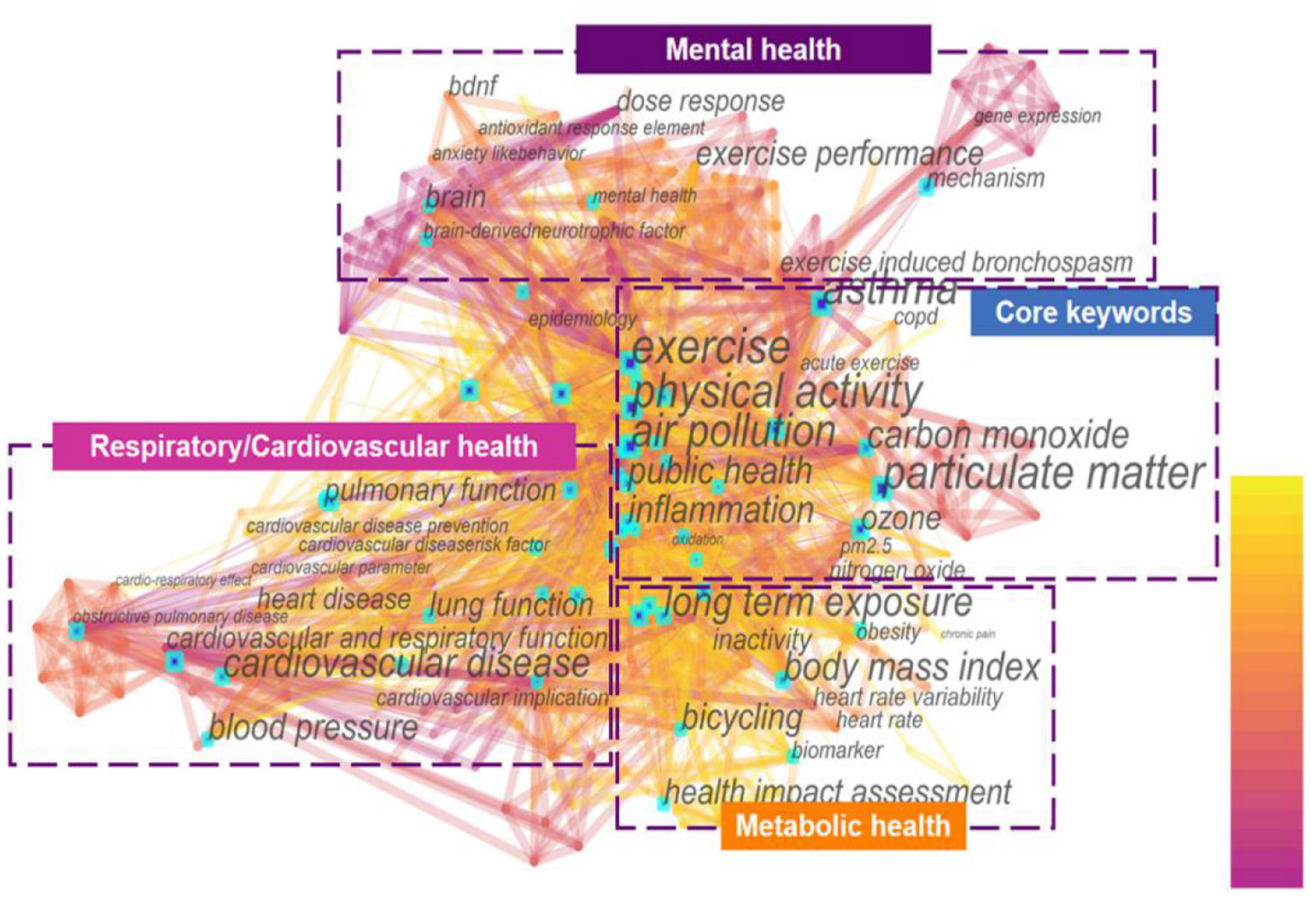
The distribution of the co-occurrence network of keywords in the AP-PE field.

A map of keywords can present major research interests and hot topics of one certain field. CiteSpace software was applied to conduct the keywords co-occurrence map (Figure 6) in this study. The merged network in this figure consisted of 420 nodes and 2,045 links. Among the more than 400 keywords, the highest frequency belonged to the “air pollution,” “physical activity,” “exercise,” which appeared 126, 90, and 61 times, respectively. This indicated that these three terms were the core subject words in the domain. It was easy to identify that the keywords “public health,” “inflammation,” and “particulate matter” have also received extensive attention.

**Figure 7:**
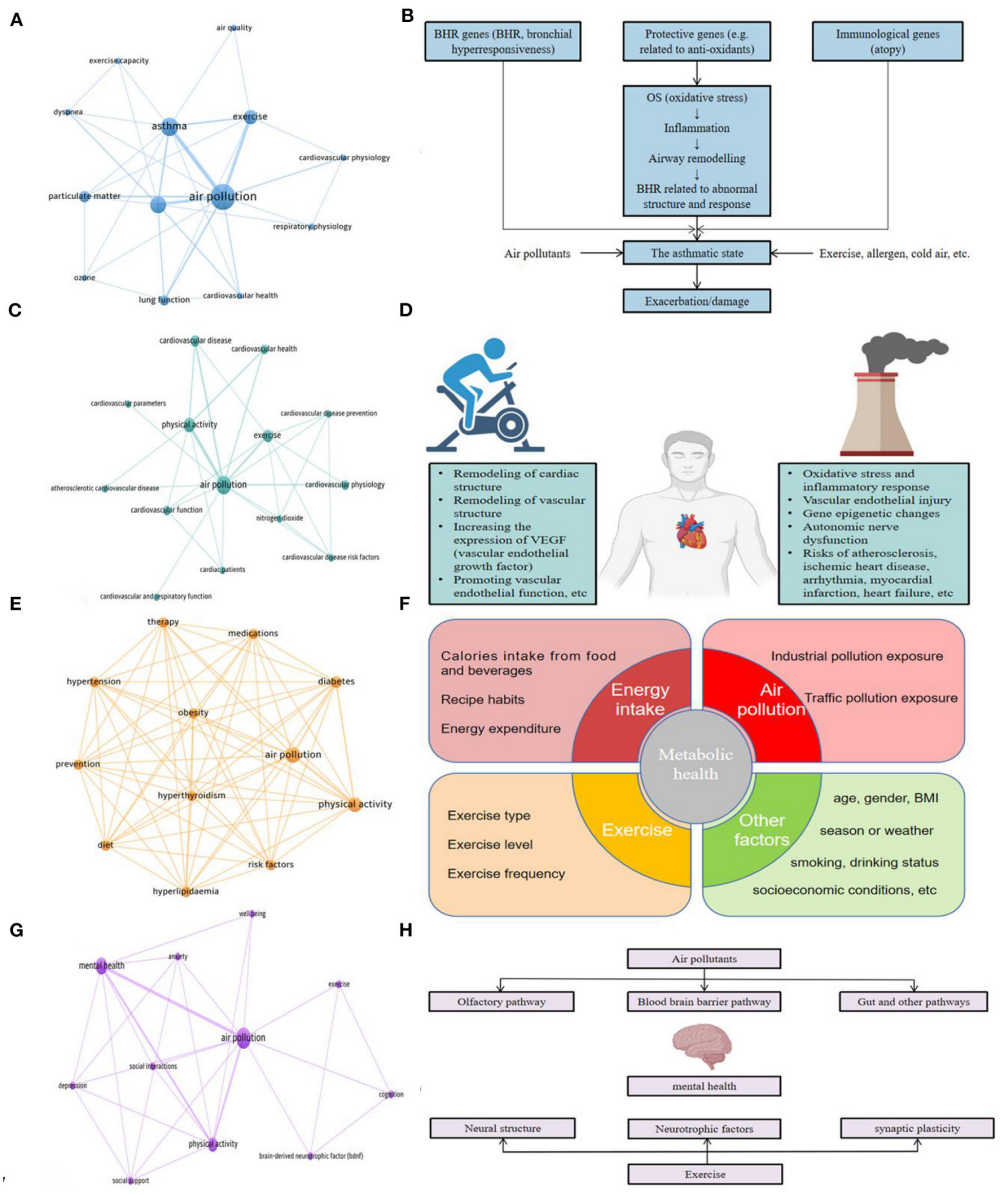
**(A)** Network connections among keywords in respiratory health-related research in the AP-PE field; **(B)** the mechanistic process for air pollutants leading to asthma **(B)** was adapted from reference Gowers et al. (2012); **(C)** network connections among keywords in cardiovascular health-related research in the AP-PE field; **(D)** mechanisms and relationships between exercise and air pollutants to the cardiovascular system; **(E)** network connections among keywords in metabolic health-related research in the AP-PE field; **(F)** major factors affecting metabolic health; **(G)** network connections among keywords in mental health-related research in the AP-PE field; **(H)** pathways and factors of air pollutants and exercise on mental health.

To better understand the research hotspots, the keywords of AP-PE field were divided into four clusters: “respiratory health,” “cardiovascular health,” “metabolic health,” and “mental health” for further analysis. The network distribution of keywords and its related mechanisms and factors are presented in Figure 7. Cluster “respiratory health” studied the impact of exercise on the respiratory system under air pollution conditions and the related risks it may cause, mainly about chronic obstructive pulmonary disease (COPD), asthma, and lung cancer. Cluster “cardiovascular health” focused on the related research of circulatory system as heart and blood vessels, which were represented by “cardiovascular diseases,” “myocardial infarction,” “arrhythmia,” and other risk factors. Cluster “metabolic health” introduced reports on the interaction factors of air pollution and exercise on glucose, endocrine, and insulin metabolism, which was mostly related to metabolic problems, such as type 2 diabetes (T2D), obesity, hyperlipidemia, and hypertension. The “mental health” cluster was an emerging topic that emphasized the AP-PE factors on brain and nervous systems. In this topic, “depression,” “anxiety,” and “brain-derived neurotrophic factor (BDNF)” were highly referred keywords. Based on the above analysis results, the research hotspots in the AP-PE field are discussed in depth in section Research Hotspots and Application Fields Analysis.

## Research Hotspots and Application Fields Analysis

### Respiratory Health

Obviously, the organ directly affected by inhalation of polluted air is the respiratory system. In view of this fact, we understood that the research focusing on respiratory system is the basis of the whole AP-PE domain. Figure 7A is a keyword co-occurrence presentation drawn by keywords of respiratory health-related literature from the total 181 references included.

As AP is often accompanied by slower air velocity and higher concentrations of a particulate matter, people may unconsciously inhale more the deposited particulate matter and other harmful substances when they exercise in an air-polluted environment. In addition, the humidity in the AP environment is relatively high, which hinders the alveolar gas exchange to a certain extent, resulting in insufficient oxygen supply. The airway and the respiratory system, as the body's first barrier, are exposed to higher risks and always the first to be attacked. Studies showed that 9% of inhaled fine PM was deposited in the lung with 6%, reaching the alveolar region (Venkataraman and Kao, 1999). More specifically, one report further proved that a moderate-intensity exercise (minute ventilation of 38 L/min) in air pollutants can result in a 4.5-fold increment of the fractional deposition of a particulate matter (Daigle et al., 2003). In this section, research topics about airway inflammations, asthma, chronic obstructive pulmonary disease (COPD), lung cancer, and other symptoms (Berend, 2016; Bowatte et al., 2017; Schultz et al., 2017) associated with AP-PE fields are discussed in detail.

Airway inflammation is a sign of the body's response to AP exposure (e.g., ozone, nitrogen dioxide, and PM <2.5 μm in diameter) in the early stages (Seltzer et al., 1986; Aris et al., 1993; Solomon et al., 2000; Frampton et al., 2002). Similarly, airway hyper-responsiveness is another body's defensive response to AP stimuli and damage (Seltzer et al., 1986; Poynter et al., 2006). Pollutants are inhaled through the mouth and nose, and enter the lungs along each branch of the main bronchus. During this process, air pollutants may deposit in various parts of the respiratory system, such as nasal mucosa, respiratory tract, and alveoli, and stimulate macrophages and epithelial cells to induce inflammation.

Moreover, exposure to AP has been considered as one of the main causes of asthma. Researchers found that there was a correlation between the level of atmospheric particulate matter and the hospitalization of asthmatic patients (Malig et al., 2013; Tian et al., 2017). The current mainstream held the point urbanization seemed to be the most common contributor to asthma due to the rapid growth of roadway traffic and outdoor AP (Brunekreef et al., 2009; Robinson et al., 2011; Perez et al., 2013). Gowers et al. (2012) concluded a mechanism framework of the process that AP leads to asthma, which maintained oxidative stress, inflammation, airway remodeling, and immunological genes. As shown in Figure 7B, at first stage, air pollutants cause oxidative stress, and then it could further activate the expression of inflammatory factors and awaken the immunological responses; subsequently, genes related to airway development and repair, including remodeling, regulate the process of asthma and rehabilitation, and, in this way, a wider range of pathways and biological mechanisms is involved in the interactions.

Long-term physical activities in air-polluted environment could further lead to chronic COPD and even lung cancer. COPD is a chronic airway inflammatory disease and has been a major public health problem worldwide (Lopez-Campos et al., 2016). With the increasing incidence rate and mortality of COPD, there is growing evidence that proved that AP might increase the incidence rate of COPD (Hansel et al., 2016; Xia et al., 2016). As for the lung cancer, it is one of the most common malignant tumor, which has become the leading cause of death in great number of regions. Particulate matter (PM) contains a variety of carcinogenic or cancer-promoting components, including polycyclic aromatic hydrocarbons, cadmium, and mercury. One epidemiological research also supported the associations between PM and cancer, indicating that the mortality of lung cancer increased by 8% for every 10 pg/m^−3^ increase in PM concentration (Pope et al., 2002).

In short, due to the fact that exercise in AP can induce airway and lung inflammation, and even cause asthma, chronic obstructive pulmonary disease, and lung cancer, the injury to respiratory system in AP context cannot be underestimated. Hence, in this condition, the benefits of exercise to respiratory system may be offset.

### Cardiovascular Health

Cardiovascular health is closely related to quality of life. In 2015, the global disease burden estimated that all forms of pollution combined were responsible for 21% of all deaths from cardiovascular disease (Landrigan et al., 2018). Figure 7C represents the highly referred keywords of pieces of AP-PE research on the special issue of cardiovascular health. From this figure, we can obtain that the keyword with cardio as the root appears frequently indicates that cardiovascular-related research is another interest in the AP-PE field.

A meta-analysis involved in 34 studies found that most air pollutants were significantly associated with a near-term increase in myocardial infarction risk (Mustafic et al., 2012). Another meta-analysis, which contained 59 studies, further demonstrated that every 10 μg/m^3^ increase in PM_10_ or PM_2.5_ can lead to an upward risk of cardiovascular disease by 0.36 and 0.63%, respectively (Lu et al., 2015b). Considering that repeated and uninterrupted exposure to air pollutants is regarded as one important risk factor in atherosclerosis, several regions faced greater cardiovascular health risks due to long-term exposure in air pollution (Wang et al., 2016). When it comes to the biological effects, the mechanisms of cardiovascular system injury caused by air pollutants have been partially studied, including oxidative stress, inflammatory response, vascular endothelial injury, gene epigenetic changes, and autonomic nerve dysfunction (Meng et al., 2016; Pope et al., 2016; Hamanaka and Mutlu, 2018; Rao et al., 2018). However, exercise training has been used as a supplementary therapy to treat cardiovascular diseases (Lavie et al., 2009; Fagard, 2011). It is self-evident that regular exercise and physical activities are safe, feasible, acceptable approaches to effectively improve cardiopulmonary function and reduce the risk of cardiovascular mortality (Nocon et al., 2008; Lee et al., 2014). The main mechanism of exercise improving the structure and function of cardiovascular system lies in remodeling of cardiac structure (cardiac physiological hypertrophy); improving the vascular structure (making the diameter of arterial vessels larger and the vessel wall thinner); increasing the expression of vascular endothelial growth factor (VEGF) in skeletal muscle and myocardium; and promoting vascular endothelial function, etc. (Green et al., 2008; Golbidi and Laher, 2011; Thijssen et al., 2012). A summary of the effects of exercise and air pollutants on the cardiovascular system is shown in Figure 7D.

The difficulty is that whether the benefits of exercise to cardiovascular system can be offset. Unfortunately, although there were still several doubts, the accessible evidence tended to point to this fact. An early research on traffic pollutants pointed out that exercise while commuting has an influence on inhaled PM, which was significantly associated with acute declines in heart rate variability, especially in pedestrians and cyclists (Nyhan et al., 2014). From the same perspective of traffic air pollution, another study further proved that whether exercising or not, every additional 1 μg/m^3^ of particle matter (2.5–10 μm) can reduce forced expiratory volume in 1 s (FEV1) and forced vital capacity (FVC). Moreover, one recent study in young and healthy males has also found that exposure to ambient air pollution during short-term submaximal exercise is associated with a decrease in airflow (FEV1/FVC) and goes one step further to state that the decrease is more apparent when the exercise takes place under particularly high exposure conditions (Kocot and Zejda, 2021). Recently, scholars using an animal model have further explored the effects of exercise in the air pollution context; their results showed that, despite a reduction in proinflammatory markers and an increase in markers of the anti-inflammatory pathway have been detected, these benefits were not enough to prevent the damage of particles to cardiovascular events (Olivo et al., 2021). Last but not least, for people with cardiovascular disease or lack of physical activity, reducing exercise may lead to a greater risk of aggravating related diseases (Kim et al., 2021; Raza et al., 2021). Therefore, it may also be beneficial to maintain a certain amount of exercise under low-concentration air pollution.

In brief, a large increase in physical activities in a high-pollution environment may adversely affect cardiovascular health, whereas exercise in the context of low-to-moderate levels of air pollutants seemed to be beneficial as well.

### Metabolic Health

Metabolism and the endocrine system jointly regulate human physiological activities and play an important role in human body. As shown in Figure 7E, we can discover that diabetes, obesity, and insulin metabolism are research hotspots in the metabolic-related topics in the AP-PE field. Overweight, obesity, and diabetes (especially T2D) are also leading risk factors and global public health burdens (Collaborators et al., 2017; Saeedi et al., 2019; Ampofo and Boateng, 2020). It should be noted that metabolism is a long-cycle process and can be affected by many factors. Here, we summarized the major factors that affect metabolic health, including physical activity, environment, diet habits, and other aspects (as shown in Figure 7F), including age, gender, body mass index (BMI), smoking/drinking status, season/weather, and socioeconomic conditions, etc. Based on these facts, there are few studies specifically targeting at physical activity or air pollution, and more are the comprehensive effects of complex factors on metabolism.

A global survey in 2016 showed that PM_2.5_ led to 3.2 million new diabetes cases worldwide (accounting for 14% of the total number of new added reports) (Bowe et al., 2018). The relationship between air pollution and metabolic diseases has been widely studied, and pieces of evidence from many countries and regions in China, the United States, and Italy confirmed that air pollutants can lead to increased risk of diabetes and obesity (Dales et al., 2012; Solimini et al., 2015; Yang et al., 2018; Cao et al., 2021). The mechanism between air pollution and the blood glucose level has been studied in physiological mechanism. It is suggested that a series of pathways may be involved in the pathogenesis and development of diabetes and its related disease through disturbance of glucose metabolism, increase of the blood insulin level, elevation of insulin resistance index, induction of oxidative stress, and inflammation (Sun et al., 2005; Anderson et al., 2012; Fleisch et al., 2014). Physical exercise has been proved to improve insulin sensitivity and systematic metabolism *via* multiple adaptations. These adaptations are underpinned by inter-tissue communications (including skeletal muscle, liver, fat, and other major target organs of insulin action), which can ultimately prevent metabolic derangement (Hawley and Lessard, 2008; Saotome et al., 2019; Grunewald et al., 2020; Thyfault and Bergouignan, 2020; Iaccarino et al., 2021). On the other hand, another mechanism that exercise promotes metabolic health is that skeletal muscle can secrete a variety of bioactive cytokines (also known as myokines) during or after the training process. These myokines can act on various tissues and organs of the whole body through endocrine or paracrine ways. Taking irisin as an example, it can promote the browning of white fat so as to promote heat production and energy consumption (Bostrom et al., 2012). In addition, exercise-induced mediators can adjust the cross-talk between many tissues (including liver, heart, pancreas, gut microbiota, and brain), and these adaptions are beneficial to prevent excessive inflammation and oxidative stress (McGee and Hargreaves, 2020).

At present, most studies on physical activity and metabolic health in the AP context are longitudinal studies. We assume that the possible reason is that, unlike the respiratory and cardiovascular systems, the process of human metabolism is usually an evolutionary process rather than immediate changes and results that can be observed. One longitudinal cohort study in adults showed that a high level of physical activities and low PM_2.5_ exposures were associated with a lower risk of hypertension and recommended regular PE can prevent hypertension for people residing in relatively polluted regions (Guo et al., 2020). On this basis, a recent study has been surprised to find that certain levels of physical activities can counteract the telomere length (TL) relative shortening caused by long-term exposure to NO_2_ and PMs in impaired fasting glucose (IFG) participants as well as patients with T2D mellitus (T2DM) (Li et al., 2021a). Another study focused on elder groups also suggested that engaging in 5 or more times of moderate to vigorous physical activity (MVPA)/week was associated with decreased risk of diabetes within groups with both high and low/moderate levels of exposure to air pollutants, such as PM_10_ or PM_2.5_, and concluded that MVPA may be inversely associated with the risk of diabetes with particulate matter exposure (Kim et al., 2020). In addition to longitudinal intervention, there are still a few studies on the impact of short-term exercise in air pollution on the metabolic system. A novel research (Wang et al., 2021) conducted in college students suggested that intermittent exercise in acute ozone exposure may trigger autonomic nervous system (ANS) imbalance and activate the hypothalamic-pituitary-adrenal (HPA) and sympatho-adrenomedullary (SAM) axes, and the metabolomics assay detected that associated pathways were in relation to steroid hormone metabolism, acute inflammatory response, oxidative stress, as well as energy metabolisms.

To sum up, compared with AP-PE's research on the respiratory and cardiovascular systems, the effects and mechanisms of metabolism are more complex, and the reasons for these changes need to be further revealed. In view of the fact that metabolism is a long-term and cyclic process, and it is difficult to observe substantial changes in short-term research, studies on this topic should pay more attention to the influence of possible confounding factors on the results.

### Mental Health

Central nervous system (CNS) is the high-level center that regulates human activities, and mental health has attracted more attention than ever in the new century. Surprisingly, the microscopic particles sifting from freeways and power plants not only harm the heart and lungs but also attack the brain (Underwood, 2017). Figure 7G demonstrates the hot topics of pieces of AP-PE research on the mental health. From this figure, we can acquire that co-occurrence words represented by cognition, anxiety, and depression are frequently referred themes.

Figure 7H presents the pathways and factors of air pollutants and exercise on mental health. Evidence from epidemiological perspective has shown that air pollution exposure is associated with CNS-related diseases, including Alzheimer's disease (AD) (Calderon-Garciduenas et al., 2004; Younan et al., 2020; Chen et al., 2021; Rhew et al., 2021), Parkinson's disease (Chen et al., 2017; Jo et al., 2021; Yu et al., 2021), stroke (Tsai et al., 2003; Kettunen et al., 2007; Lu et al., 2021), and depression (Zijlema et al., 2016; Li et al., 2021b; Shih et al., 2021). How do these air pollutants affect the brain system? Early studies found that there are two main ways for the transport of air pollutants into the central nervous system (Oberdorster et al., 2004; Lampron et al., 2013): one way is that air pollutants precipitate in the respiratory tract and transport to the blood circulation system, and then penetrate the blood-brain barrier (BBB) into the brain; another approach is to pollutants deposit on the nasal and enters the CNS through the olfactory bulb of the brain. Despite the above two pathways, recent studies have also detected that air pollutants might affect the intestinal microbiome and interfere with the brain-gut axis pathway, resulting in neuro inflammation (Mutlu et al., 2011, 2018; Shou et al., 2019). Furthermore, pollutants entering the brain can cause nerve damage, and its main mechanisms include oxidative stress (Calderon-Garciduenas et al., 2015; Verma et al., 2015) and inflammatory response (Ku et al., 2017). In addition, studies have shown that particulate matters can lead to neurotoxicity by mediating epigenetic regulation (Gondalia et al., 2019). However, previous studies have shown that exercise is not only beneficial to improve the brain function of healthy people but also has a protective and preventive effect on the patients with neurological diseases such as AD and depression (Heyn et al., 2004; Lautenschlager et al., 2008; Wegner et al., 2014; Kvam et al., 2016). The major effects of exercise on mental health can be divided into three aspects: remodeling of neural structure (Voss et al., 2013; Bonavita and Tedeschi, 2017), generation of neurotrophins (Dishman et al., 2006; Liang et al., 2021), and improvement of synaptic plasticity (Dishman et al., 2006; Petzinger et al., 2013; Liang et al., 2021). It should be noted that brain-derived neurotrophic factor (BDNF) is one of the key factors in improving cognition and mental health mediated by exercise. Studies have shown that impaired cognitive function and psychiatric disorders are closely related to the decline of the BDNF level (Connor et al., 1997; Shimizu et al., 2003; Angelucci et al., 2005; Cramer and Riley, 2008), and exercise can significantly upregulate the expression of BDNF and glial cell-derived neurotrophic factor (GDNF) (Aguiar et al., 2014; Soke et al., 2021). Besides, there are also studies that show that exercise can promote the enhancement of mitochondrial adaptability, and then improve the brain oxidative stress environment (Navarro et al., 2004; Gomez-Pinilla, 2008; Gusdon et al., 2017; Gan et al., 2018) so as to prevent or rehabilitate related neuropsychiatric diseases.

So far, there is shortage of in-depth studies on the interactive effects of AP and exercise on brain function. We preliminarily suppose that the reasons for this are not only the complexity of basic exploration in brain science itself but also the fact that changes in brain structure and function usually take time to accumulate. One short-term study explored the effects of exercise in different environments, while the results indicated that one 15-min bout of walking or jogging with or without air pollutants did not appear to affect participants' emotions (Han, 2020). There are also studies using aerobic and resistance training under the air pollutants conditions on cognition status (Molina-Sotomayor et al., 2019, 2020), and their findings tended to show that exercise may be serve as a protective factor against the effects that pollution has on cognition. However, most of these studies use evaluation methods such as blood parameters or mental scales, and there is little research on the mechanism of cognitive function changes. On the basis of these facts, several studies tried to answer the questions with the application of animal models, and it seemed that ultrafine particles exposure might mitigate the benefits of the exercise-induced upregulation of BDNF gene expression in the rats' hippocampus (Bos et al., 2012a,b). Additionally, changes in gene or mRNA levels are not necessarily consistent with protein levels (Soya et al., 2007), so these findings need to be confirmed on further dimensions.

In a nutshell, brain research and mental health have been emerging topics, also the crucial and difficult issues, in recent AP-PE studies. The balance between neurotoxicity caused by air pollutants and neurotrophic mechanism induced by exercise is not clear. What is certain is that BDNF is a mediator connecting air pollution and exercise-induced changes in brain function. Taken BDNF and its related neurotrophic factor as a bridge, further research can explore more molecular events and specific pathways under the AP-PE context.

## Conclusion and Future Perspective

### Summary

For the first time, the research distribution, major topics, and relevant hotspots of the AP-PE field have been described by the knowledge-map strategy. Despite previous articles that have been conducted on the cohort design, associated mechanisms, interaction factors, etc., the understanding of total exercise and physical activities in an air-polluted environment is still limited. One reason is that exercise, as an emerging active living style, has mushroomed in recent years, and environmental health has attracted increasing attention since the new century. Another may be the development history of AP-PE interdisciplinary is relatively short, although the analysis of annual outputs and subjects suggests that it is currently in the prosperity era of the research field.

In this study, a total of 181 studies in the AP-PE field were retrieved from the WoSCC. The trend of annual publications displayed notable growth overall, especially from 2016 to present. Most of the related pieces of research were published in the journals with a focus on environment, medicine, and sports. The research contexts were mainly involved in commuter exposure, urban environmental health, public transportation, health impact assessment; the effects and modes are mostly connected with exercise immunology, short-term exposure, and moderate aerobic exercise, and the noteworthy subjects included youth, elite athletes, sports facilities, and rural population. In addition, on the basis of the AP and exercise's effects on different aspects of the body, we further discussed the four application fields in depth. Respiratory system, as the first barrier of the body to defense against air pollutants, has been widely studied, and it seems that the benefits of exercise to respiratory system may be offset under AP conditions. There are debates on the AP-PE condition to the cardiovascular health, and the AP level is an important factor mediating the beneficial effects of physical exercise. Metabolic pieces of research in AP-PE fields are closely related to public health issues, including diabetes, obesity, hypertension, etc. Since metabolism is a mixed result regulated by multiple axes, the metabolic studies need to be further explored with confounding factors included. Brain science, as the focus and emerging field of pieces of AP-PE research, is springing up nowadays. BDNF seems to be a link between exercise benefits and brain impairments caused by air pollution, while more mechanisms at the molecular and pathway levels remain to be discovered in the upcoming years.

### Future Perspectives

According to the knowledge map and visualized results in our study, we consider that future research should pay more attention to the following concerns:

Overall, the AP-PE studies are still in the development stage and remained to be further standardized and systematized. In current studies, the differences in the research paradigm, sample size, exposure method, model selection, and outcome detection may lead to inconsistent research conclusions and difficult to integrate. To provide a greater scope for improvement, it is urgent to establish a scientific standard and conclude biomarkers and major indicators to guide future scholars through the directions to more explicitly explore the field. In this study, we summarized that the current mainstream research strategies are mostly related to the five aspects: (1) longitudinal studies on physical exercise exposed to long-term air pollution, typically represented by traffic pollution context. This type mainly focuses on the mitigation of benefits induced by exercise under air pollution. (2) Short-term intervention under one acute exercise experience under air pollution condition. This mode is mostly used to evaluate the exercise performance of elite athletes in special situations, such as Olympic Games and other sporting occasions. (3) Long-term empirical research that contains pre-exercise training in a non-exposed environment, and then comparing the trained groups' and non-trained groups' health status in air pollution. This study design is generally applicable in regions with intermittent air pollution and aims to observe whether the protective effects of exercise (including the improvement of immunity) can reduce the damage to the body caused by air pollution. (4) Different from the subjects selected in the first three modes, the fourth type of research tends to select patients living in air-polluted areas with basic chronic diseases as the control group, and explore if exercise can rehabilitate or improve air pollution-related diseases to some extent. (5) Pieces of research combined with two or more of the above four modes.

Although numerous AP-PE studies have detected the oxidative stress and inflammatory response on health system, few have focused on underlying mechanisms. When the focus is the composition of air pollutants, varied sources of pollutants in different regions and seasons are complex; thus, the components inducing toxicological effects are not well-understood. The molecular mechanism research on the components of major pollutants (such as PM) will help researchers to explain the impact of specific pollutants on body health, and, as well, has important guidelines for AP control. Additionally, previous reports mainly focus on the partial effect of one organ, and the research on the integration mechanism of AP, exercise, and health is still missing. Actually, the effects of pollutants and exercise on the body are systematic. For example, the stress reaction is related to the HPA axis induced by brain, and intestinal brain carries out negative feedback regulation of intestinal flora *via* the brain-gut axis, etc. Therefore, with the establishment and maturity of hybrid research strategy and multi omics technology, the research based on phenotype-function and upstream-downstream effectors will provide a new horizon for scholars to understand the profound mechanism under the AP-PE context.

Furthermore, one of the main challenges for pieces of AP-PE research regarding the dose relationship is to explore the dose effect of exercise under air pollution. There are two equilibrium points in the yield curve of AP-PE activities. One is the intensity and the mode of the exercise; another is the level and the component of air pollutants. Although there are some ongoing studies on the dose response between exercise and AP, the evidence level is not sufficient to support the explanation of the associated phenomenon. Up to now, the consensus on exercise is that low-to-medium intensity has a positive effect on reducing metabolism risks and improving the aerobic capacity, while high-intensity exercise has a better effect on the enhancement of cardiopulmonary fitness. Hence, it needs to be further clarified which exercise mode and intensity will bring better health effects under the AP context. Based on the acquired knowledge, it seems that doing one-bout aerobic exercise (a power bicycle) during an air quality index (AQI) ranking of “yellow” will not diminish exercise performance in healthy adults, nor has a negative effect on pulmonary function or biological health markers (Wagner and Clark, 2018). There is also evidence that the beneficial effects during exercise will be mitigated in PM_2.5_ > 100 μg/m^3^ (Tainio et al., 2016; Pasqua et al., 2018), which indicates that it should not be encouraged to exercise if PM_2.5_ is greater than or equal to this value. Simultaneously, when the existing research cannot provide accurate physical activity guidelines under light or mild air pollution conditions, our recommendation for the exercise strategy is “listening to the body,” since the intensity of activity that allows the body to adapt to air pollutants might be safe in these cases.

Totally speaking, even with increased amount of AP-PE publications in recent years, further research is still required to (i) establish the framework and standard to clarify the key issues and answer the major concerns; (ii) strengthen the interdisciplinary collaborations, and explore the systematic impact of molecular mechanisms and specific pathways on the whole life cycle and multi-organ crosstalk; (iii) find out the equilibrium points as well as the aspects of dose-effect relationships between exercise and the AP level.

### Strength and Limitations

The greatest strength of our study lies in the extensive analysis of global publications on PE and AP conditions from an interdisciplinary perspective and novel strategy. The combination of the knowledge map and visualized analysis can quantitatively reflect research status and practical applications simultaneously, and demonstrate the distribution of collaborations among countries, regions, disciplines, etc. Our study applies the structured information, including clusters of co-cited references and keywords, while the use of unstructured knowledge and qualitative approaches is limited. Subsequently, we only use the four indexes of Web of Science Core Collection for analysis while the document included in one database may not be comprehensive. However, the realistic dilemma is that the existing knowledge map software does not support the extraction and meta-analysis of multi-database records. In addition, since the research included in this paper is not comprehensive enough on specific components of air pollutants, the relationship between multiple pollutants and physical activities remains to be further discovered. Based on our work, future scholars can use artificial intelligence combined with multiple-database analysis to further explore a research development path and promising aspects in the field of physical exercise or activity under the air pollution conditions.

## Author Contributions

Conceptualization was contributed by YY, WL, and XM. Methodology was contributed by YY, DW, and YC. Validation was contributed by YY, DW, and JL. Formal analysis was contributed by YY, JL, and YC. Writing—original draft preparation was contributed by YY. Writing—review and editing were contributed by YY and DW. Supervision was contributed by WL and XM. Project administration was contributed by WL and XM. Funding acquisition was contributed by XM. All the authors have read and agreed to the published version of the manuscript.

## Funding

This work was supported by National Social Science Fund of China under Grant No. 16BTY065 and Leading Talents of Independent Research Program of Tsinghua University under Grant No. 2016THZWLJ12.

## Author Disclaimer

The views and ideas expressed herein are solely the authors' and do not represent the ideas of the funding agencies in any form.

## Conflict of Interest

The authors declare that the research was conducted in the absence of any commercial or financial relationships that could be construed as a potential conflict of interest.

## Publisher's Note

All claims expressed in this article are solely those of the authors and do not necessarily represent those of their affiliated organizations, or those of the publisher, the editors and the reviewers. Any product that may be evaluated in this article, or claim that may be made by its manufacturer, is not guaranteed or endorsed by the publisher.
